# A community proposal to integrate structural bioinformatics activities in ELIXIR (3D-Bioinfo Community)

**DOI:** 10.12688/f1000research.20559.1

**Published:** 2020-04-22

**Authors:** Christine Orengo, Sameer Velankar, Shoshana Wodak, Vincent Zoete, Alexandre M.J.J. Bonvin, Arne Elofsson, K. Anton Feenstra, Dietland L. Gerloff, Thomas Hamelryck, John M. Hancock, Manuela Helmer-Citterich, Adam Hospital, Modesto Orozco, Anastassis Perrakis, Matthias Rarey, Claudio Soares, Joel L. Sussman, Janet M. Thornton, Pierre Tuffery, Gabor Tusnady, Rikkert Wierenga, Tiina Salminen, Bohdan Schneider

**Affiliations:** 1 Structural and Molecular Biology Department, University College, London, UK; 2 Protein Data Bank in Europe, European Molecular Biology Laboratory, European Bioinformatics Institute, Hinxton, CB10 1SD, UK; 3 VIB-VUB Center for Structural Biology, Brussels, Belgium; 4 Department of Oncology, Lausanne University, Swiss Institute of Bioinformatics, Lausanne, Switzerland; 5 Bijvoet Center, Faculty of Science – Chemistry, Utrecht University, Utrecht, 3584CH, The Netherlands; 6 Science for Life Laboratory, Stockholm University, Solna, S-17121, Sweden; 7 Dept. Computer Science, Center for Integrative Bioinformatics VU (IBIVU), Vrije Universiteit, Amsterdam, 1081 HV, The Netherlands; 8 Luxembourg Centre for Systems Biomedicine, University of Luxembourg, Belvaux, L-4367, Luxembourg; 9 Bioinformatics center, Department of Biology, University of Copenhagen, Copenhagen, DK-2200, Denmark; 10 ELIXIR Hub, ELIXIR, Hinxton, UK; 11 Department of Biology, University of Rome Tor Vergata, Rome, I-00133, Italy; 12 Institute for Research in Biomedicine, The Barcelona Institute of Science and Technology, Barcelona, 08028, Spain; 13 Netherlands Cancer Institute and Oncode Institute, Utrecht, The Netherlands; 14 ZBH - Center for Bioinformatics, Universität Hamburg, Hamburg, D-20146, Germany; 15 Instituto de Tecnologia Química e Biológica Antonio Xavier, Universidade Nova de Lisboa, Lisbon, Portugal; 16 Department of Structural Biology, Weizmann Institute of Science, Rehovot, 76100, Israel; 17 European Molecular Biology Laboratory, European Bioinformatics Institute, Hinxton, CB10 1SD, UK; 18 Ressource Parisienne en Bioinformatique Structurale, Université de Paris, Paris, F-75205, France; 19 Membrane Bioinformatics Research Group, Institute of Enzymology, Budapest, H-1117, Hungary; 20 FBMM, Biocenter Oulu, University of Oulu, Oulu, Finland; 21 Structural Bioinformatics Laboratory, Åbo Akademi University, Turku, FI-20500, Finland; 22 Institute of Biotechnology of the Czech Academy of Sciences, Vestec, CZ-25250, Czech Republic

**Keywords:** structural bioinformatics, biomolecular structure, protein structure, nucleic acids structure, ELIXIR, Instruct-ERIC

## Abstract

Structural bioinformatics provides the scientific methods and tools to analyse, archive, validate, and present the biomolecular structure data generated by the structural biology community. It also provides an important link with the genomics community, as structural bioinformaticians also use the extensive sequence data to predict protein structures and their functional sites. A very broad and active community of structural bioinformaticians exists across Europe, and 3D-Bioinfo will establish formal platforms to address their needs and better integrate their activities and initiatives. Our mission will be to strengthen the ties with the structural biology research communities in Europe covering life sciences, as well as chemistry and physics and to bridge the gap between these researchers in order to fully realize the potential of structural bioinformatics. Our Community will also undertake dedicated educational, training and outreach efforts to facilitate this, bringing new insights and thus facilitating the development of much needed innovative applications e.g. for human health, drug and protein design. Our combined efforts will be of critical importance to keep the European research efforts competitive in this respect.

Here we highlight the major European contributions to the field of structural bioinformatics, the most pressing challenges remaining and how Europe-wide interactions, enabled by ELIXIR and its platforms, will help in addressing these challenges and in coordinating structural bioinformatics resources across Europe. In particular, we present recent activities and future plans to consolidate an ELIXIR 3D-Bioinfo Community in structural bioinformatics and propose means to develop better links across the community. These include building new consortia, organising workshops to establish data standards and seeking community agreement on benchmark data sets and strategies. We also highlight existing and planned collaborations with other ELIXIR Communities and other European infrastructures, such as the structural biology community supported by Instruct-ERIC, with whom we have synergies and overlapping common interests.

## List of abbreviations

3D-Bioinfo: name of the ELIXIR Community of structural bioinformatics

BioExcel: Center of excellence for biomolecular research

Biomedinfra: authenticaton and authorisation infrastructure (ELIXIR AAI) of ELIXIR Finland

CAMEO: Continuous Automated Model Evaluation

CAPRI: community-wide experiment on the comparative evaluation of protein-protein docking for structure prediction

CASP: critical Assessment of protein structure prediction

ChEMBL: a manually curated database of bioactive molecules with drug-like properties

COOT: crystallographic object-oriented toolkit, a graphics for refinement of experimental biomolecular structures

COST: (European) cooperation in science and technology

EMDB: Electron microscopy data bank

ELIXIR: intergovernmental organisation that brings together life science resources from across Europe

EM, cryo-EM: electron microscopy, cryo-electron microscopy

EOSC-Hub: European Open Science Cloud

EU-OPENSCREEN: integrates high-capacity screening platforms throughout Europe

FAIR: data which meet principles of findability, accessibility, interoperability, and reusability

FARFAR: fragment assembly of RNA with full-atom refinement

FARNA,: fragment assembly of RNA

Instruct, Instruct-ERIC: pan-European research infrastructure in structural biology

MD: molecular dynamics

microED: electron micro-crystallography

MMB: MacroMolecularBuilder. A program suite for macromolecular modelling

MX macromolecular X-ray crystallography

NMR: nuclear magnetic resonance, a spectroscopic method

OpenEBench: infra-structure designed to establish a continuous automated benchmarking system for bioinformatics

PDB: Protein data bank

PDBe-KB: Protein data bank in Europe - knowledge base

PDB-Redo: procedure to optimise crystallographic structure models

Phenix: software suite for the automated determination of molecular structures

PHYRE: automatic fold recognition server for predicting the structure and/or function of the protein sequence

Proteopedia: wiki and 3D encyclopedia of proteins and other biomolecules

Pubchem: database of chemical molecules and their activities

Refmac: program for refinement of experimental structures of biomolecules

RNA Puzzles: collective experiment for blind RNA structure prediction

SAXS: small angle X-ray scattering

SBDD: structure-based drug design

Swiss-model: structural bioinformatics web-server dedicated to homology modelling of 3D protein structures

TeSS portal: ELIXIR's training portal

UniProt: Universal protein esource, a comprehensive resource for protein sequence and annotation data

Web-Beagle: web server for the alignment of RNA secondary structures

## Major European contributions in structural bioinformatics

**Figure 1.**
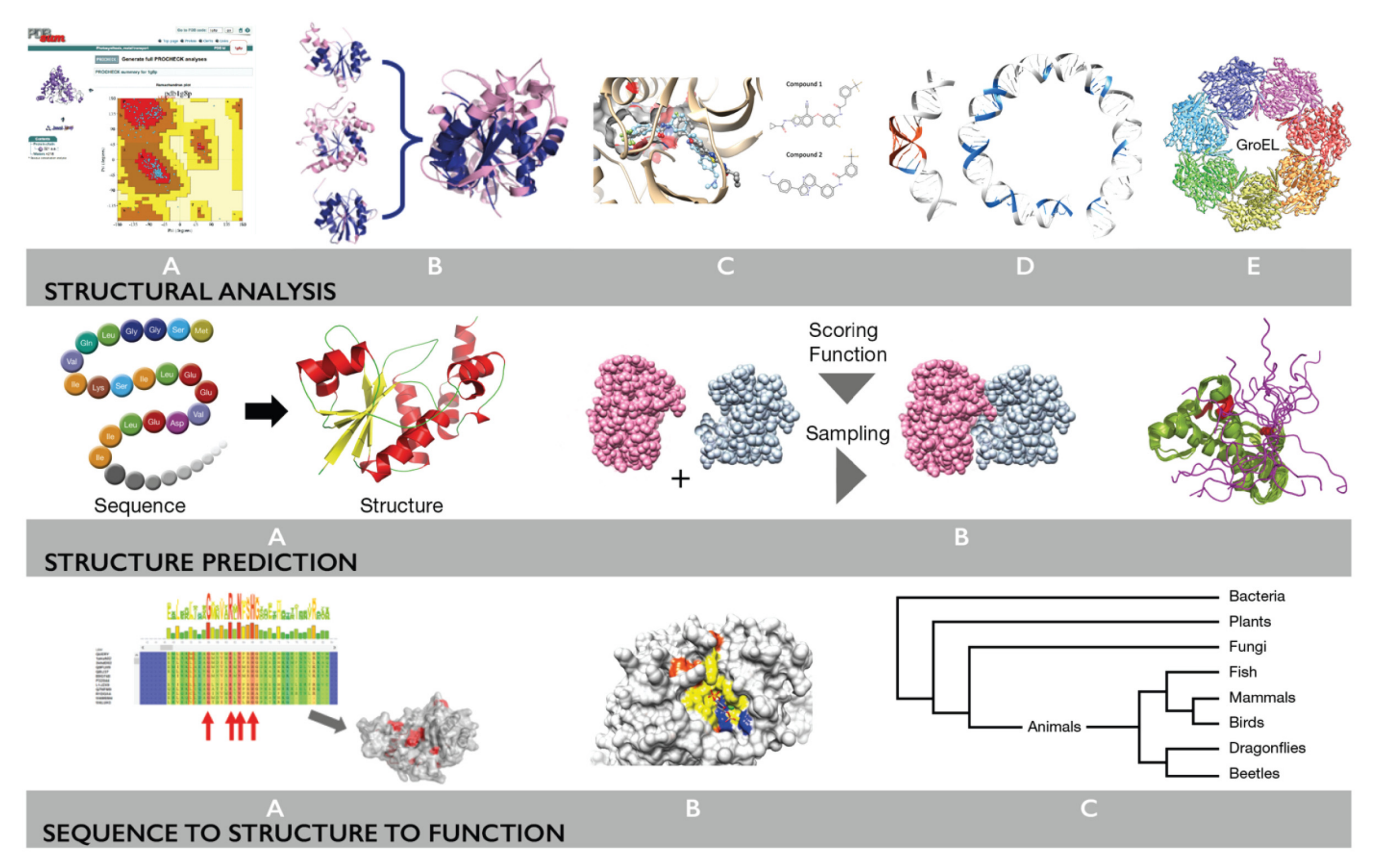
Schematic illustrating major themes in structural bioinformatics. Top row – protein structure validation, protein structure comparison and classification, protein ligand interactions, nucleic acid structures, protein-protein interactions and complexes; middle row – protein structure prediction, prediction of protein interactions, protein structure dynamics; bottom row – integration of protein structure and sequence to predict functional sites and effects of genetic variations, exploiting protein structure annotations for comparative genome studies.

Structural bioinformatics is a well-established scientific activity, which started in the 1970s following the establishment of the Protein Data Bank ^1^ which provides open access to macromolecular structure models. Figure 1 illustrates major themes in structural bioinformatics. Structure data can give deeper insights into the mechanism of proteins, the functions of biomolecules (proteins, nucleic acids, carbohydrates, lipids, etc.) and their interactions with each other and with chemical modulators of their functions (inhibitors, activators, co-factors, etc.). This enables the design of new experiments to study the function of macromolecules as well as rational design of proteins and drugs, to modify their function and properties.

**Figure 2.**
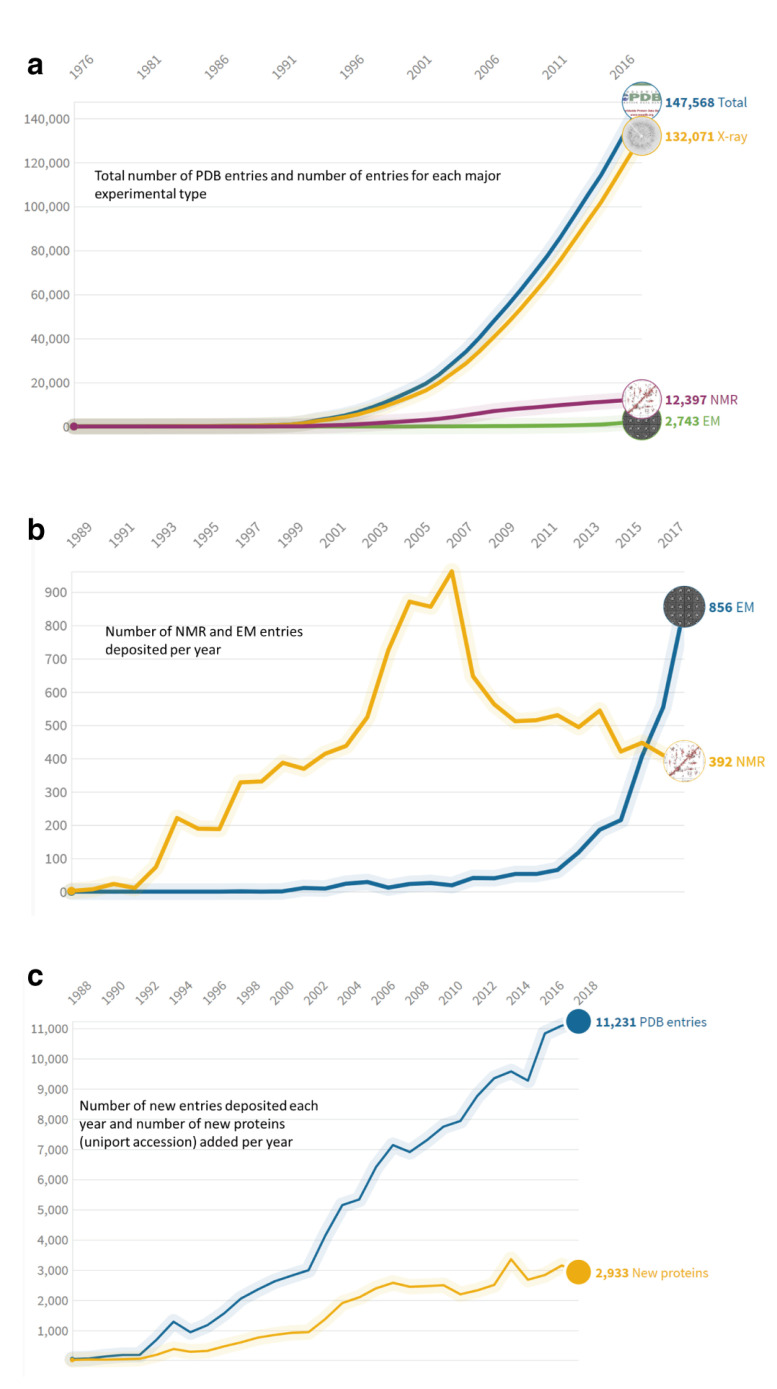
( **a**) Total number of PDB entries and number of entries for each major experimental type. ( **b**) Number of nuclear magnetic resonance (NMR) and electron microscopy (EM) entries deposited per year. ( **c**) Number of new entries deposited each year and number of new proteins (UniProt accession) added per year.

Structure models are experimentally determined by macromolecular X-ray crystallography (MX) and small angle X-ray scattering (SAXS), nuclear magnetic resonance spectroscopy (NMR), or cryo-electron microscopy (EM). The technological developments in MX in the previous decade, largely catalysed by the structural genomics initiatives and the on-going revolution in the field of cryo-EM, are expanding the volume of structural data both quantitatively and qualitatively (see Figure 2). European structural bioinformatics groups have played a crucial role in the development of methods to validate this data ^2, 3^ and the world-wide adoption of these tools by the structural biology community. They have also initiated collaborations and joint activities between structural bioinformaticians and structural biologists, expanding recently to meet the need to develop tools and validation protocols for structures determined using new techniques such as EM (cryoEM, microED) and Integrative/hybrid methods.

European bioinformatics groups have also been at the forefront of efforts to compare protein structures and characterise their features in order to understand the underlying principles of protein structure and function ^4^ and thereby promote both fundamental and translational research. For example, characterisation of protein binding pockets provided vital information for rational drug design. Europe also pioneered the establishment of comprehensive structure-based protein classifications ^5, 6^ giving structural insights into protein evolution and European structure-based tools have facilitated enzyme reaction mechanism studies by chemists and biochemists.

Another major European activity, the prediction of protein structures from amino acid sequences, started in the late 1980s. European groups were amongst the first to predict protein secondary and tertiary structure for soluble and membrane associated proteins. Additionally, some of the most critical contributions to building protein 3D models from structural templates of homologous proteins, happened in Europe in the 1990s ^7, 8^, together with the development of methods for assessing model quality. European bioinformatic groups have also provided key solutions for the hardest task of *de novo* prediction of protein spatial structures ^9^. Furthermore, European groups have made seminal contributions to the development of methods for modelling the 3D structure of protein complexes ^10^, a very difficult problem, which is centre stage for today’s molecular biology. These activities have been further expanded in the field of multi-scale modelling where a wide variety of experimental and bioinformatics data are integrated into the modelling process. Importantly European groups have made major contributions to initiatives assessing the performance of structure prediction and protein docking methods (see reviews 11– 13).

European research groups contributed significantly to the field of RNA bioinformatics, setting standards in RNA structure predictions, modeling, and data format ^14, 15^. In particular, the RNA-Puzzles experiment for evaluation of RNA structure prediction methods, and a series of associated workshops have been introduced in Europe, attracting the top groups world-wide ^16, 17, 18^.

Protein function is strongly related to molecular recognition of small molecules such as substrates, inhibitors, or signalling compounds and many European groups have been active in this area over the last 50 years ^19, 20, 21^ and remain major players in the field. Europe also has an exemplary track record in developing molecular dynamics (MD) simulation techniques and applying them to investigate dynamic properties of protein systems, functionally important conformational transitions in proteins, as well as folding and unfolding reactions ^22, 23, 24^, providing crucial insight into dynamics aspects that are notoriously difficult to capture by experimental approaches.

Protein structural data and functional residue annotations also inform protein engineering, another important activity with significant European representation. For instance, the discovery of canonical conformations in antibody variable domains ^25^ spurred the development of the first methods for accurate structure prediction in antibodies ^26^. Other biocomputational methods have been important for enzyme engineering. Such contributions by European bioinformaticians have transformed the face of protein engineering and were the basis for establishing major biotechnological companies for developing new research and clinical tools.

## Major challenges that 3D-Bioinfo will help to address

**Figure 3.**
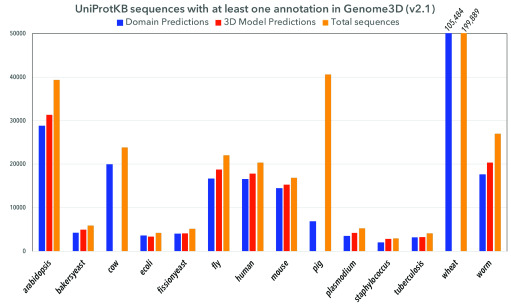
The coverage of protein sequences from selected model organisms with structural annotations provided by the Genome3D resource.

Improvements in structure prediction opens up huge possibilities including understanding the effects of disease causing mutations, and provides an essential platform for almost all future translational efforts including developing novel drugs. Furthermore, international initiatives (i.e. CASP ^27^, CAMEO ^28^ and CAPRI ^29, 30^ for assessment of the prediction of protein structures and complexes have driven the field by independently validating methods and highlighting innovations that increase performance. However, many challenges still exist. It remains computationally expensive to build 3D models on a proteome-wide scale. Furthermore, prediction methods are still error prone. It is therefore important to increase coverage and confidence measures by consolidating results from multiple methods. ELIXIR is already supporting some Europe-wide collaborative initiatives. For example, a recent implementation study links several major structure prediction and annotation resources (SWISS-MODEL ^31^, PHYRE ^32^, GenTHREADER ^33^, Fugue ^34^, SUPERFAMILY ^35^, CATH-Gene3D ^36^) with ELIXIR Core Resources, PDBe ^37^ and InterPro ^38^ to increase the coverage and reliability of predicted protein structure data (see Figure 3).

Structural bioinformatics tools link sequence and structure data to predict protein functional sites. As for protein structure prediction, integration of data on sites predicted by different methods will increase both coverage and accuracy. In this context, new initiatives like the PDBe Knowledgebase (PDBe-KB) are integrating data from multiple European groups allowing easy access, development of meta-predictors and common benchmarking to improve accuracy. Since some disease-associated genetic variations result in modifications of protein residues in or near functional sites, these initiatives provide a natural link with the ELIXIR Human Rare Disease Community.

Recent and future technological challenges of structural biology such as EM, serial crystallography, fragment screening, bio-SAXS, time-resolved structural methods, and techniques of integrated biology in general, are important areas that can be addressed by structural (3D) bioinformatics, albeit always in close collaboration with structural biology research groups. Optimal data formats, FAIRness ^39^ of the data, interoperability of the data and software tools are serious issues that require close collaboration between structural biologists and bioinformaticians.

With regard to prediction of protein-ligand interactions, protein/drug design, and modelling of dynamic properties of proteins and their interactions, much work remains to be done in benchmarking of methods and better integration of methods and data. 3D-Bioinfo will endeavour to facilitate collaborations and new initiatives in these areas.

## Goals of 3D-BioInfo

The major goals of 3D-Bioinfo will be to increase interoperability between resources by developing and promoting data standards, integrating data where appropriate and developing robust benchmarking strategies for prediction algorithms (e.g. protein structures, complexes, ligand/drug docking). We will also develop better visualization frameworks for protein and nucleic acid structures and work closely with the structural biology community and initiatives such as Instruct-ERIC to develop improved validation metrics for nucleic acid structures, an important area, which is currently underdeveloped.

The 3D-Bioinfo major goals can be summarized as follows:

• Promote and develop data standards to drive data integration

• Plan the long-term sustainability for key computational tools and data resources

• Drive the integration of resources and tools for analysis of structural data

• Develop robust standard methods for benchmarking and validating prediction tools

• Facilitate the access of tools requiring high compute power to the appropriate facilities

• Improve integration of structural data with other quantitative biological data

• Pool and expand the available training and outreach material in structural bioinformatics.

## Links between 3D-Bioinfo and the wider European research environment

The structural bioinformatics community forms an indispensable interface between producers of structural data and their users. There are already several ELIXIR endorsed node resources/servers (SWISS-MODEL ^31^, Phyre ^32^) and Core Data Resources (PDBe ^37^, CATH ^36^) in 3D-Bioinfo.

European groups participating in 3D-Bioinfo have been involved in European networks providing derived structural data and analysis tools to biologists (e.g. InteGr8, IMPACT, Biosapiens, Instruct-ERIC, BioExcel, Biomedinfra, EU-OPENSCREEN, EOSC-Hub) and COST Actions (e.g. CA15135 - Multi-target paradigm for innovative ligand identification in the drug discovery process; CM1306 - Understanding Movement and Mechanism in Molecular Machines, COST Action BM1405 (CA17139 EUTOPIA (EUropean TOPology Interdisciplinary Action), WP3 Entangled and Self-entangled Proteins). Other international collaborations include the Protein Structure Initiative (PSI) for structural genomics. A significant fraction of the tools/resources provided by ELIXIR nodes relates to structural bioinformatics and the combined resources receive hundreds of thousands of web-accesses/month from the wider global research community.

### 3D-Bioinfo Executive and Steering Committees

The 3D-Bioinfo Community includes leading figures in the field of structural bioinformatics across Europe. It was initiated in March 2018 with the formation of a Steering Committee and an Executive Committee comprising representatives from 16 ELIXIR nodes. These members actively sought further participants from within their nodes, covering a wide spectrum of skills and needs (see European groups participating in 3D-Bioinfo). They are responsible for the development and sustainable operation of complex tools ranging from databases to infrastructures promoting interoperability between multiple areas of research that rely on the reusability of structural data provided by the structural biology community (e.g. as represented by Instruct-ERIC).

### Description of the launch meeting

3D-BioInfo was launched at a meeting in Basel, September 2018, with 70 participants from 15 European countries providing their input. In addition to presentations from committee members on the proposed 3D-Bioinfo Activities, representatives of the five ELIXIR platforms on data, tools, interoperability, compute and training, described their activities. John Hancock also gave a presentation as Coordinator of ELIXIR Communities and representative of the ELIXIR hub. Additionally, 19 ‘flash talks’ were given by European research groups interested in contributing to one or more of the proposed 3D-Bioinfo activities and/or to joint activities with the ELIXIR platforms. Organisers of related ELIXIR communities (proteomics, intrinsically disordered proteins and human copy number variation) gave presentations indicating possible areas of future collaboration. There were also oral contributions from representatives of other European ESFRI initiatives like Instruct-ERIC and BioExcel, again highlighting synergies and possible collaborations. Posters from many research groups, keen to participate in 3D-Bioinfo, were presented at the meeting highlighting their possible contributions and an interactive session was held allowing participants to discuss the proposed Activities and suggest future Activities. Participants also discussed ideas for interaction with the ELIXIR platforms.

## Activities and action plans prioritized by 3D-Bioinfo

Following their formation in March 2018, the 3D-Bioinfo Executive and Steering Committees held regular conference calls to determine the highest priority areas, which would become the main Activities to be first undertaken by the community. These 3D-BioInfo Activities, outlined below, were further discussed and refined at the launch meeting, and involve multiple participating groups across the nodes.

Structural Bioinformatics is well established, as the field began more than 40 years ago following the establishment of the protein structure databank in the 1970s, so most of these Activities represent mature areas of research, some of which had already received sustained node funding to develop their tools and resources. Each Activity will have the major goals, listed above, as the core of their mission. They are being coordinated via regular conference calls and are each evolving specific tasks. Below we detail, the specific thematic aims of each Activity and highlight the planned interactions with the ELIXIR platforms.

### Activity I: Infrastructure for FAIR structural and functional annotations

***Coordinator: Sameer Velankar.*** The Protein Data Bank (PDB) and the Electron Microscopy Data Bank (EMDB) both follow the FAIR principles, thus enabling many niche data resources to derive added value annotations from the archived structural data, such as structure domain classifications, information on ligand- and macromolecule binding sites and effects of mutations on structure and function. However, a lack of coordination between these specialist data resources has prevented the creation of data standards and uniform data access mechanisms, consequently reducing the impact of these valuable data.

The proposed Activity will address these omissions by further developing the PDBe Knowledge Base ( PDBe-KB) – a community-driven data resource for structural and functional annotations that places structural data in its biological context. PDBe-KB increases the visibility and interoperability of niche data resources by collating minimally required common structure-based annotation data in a standardized data exchange format and by enabling comparisons between specific types of annotations obtained from different software tools.

The initial focus of this Activity will include three major goals: i) continue to expand the scope of integrating known and predicted functional site annotations with PDBe-KB; ii) integration of annotations concerning the impacts of disease-associated variants on structure and function in PDBe-KB; and iii) integration of predicted protein structure data via existing model archives by establishing a federated infrastructure for access (3D-Beacons).

*Identifying additional predicted or manually curated annotations that could further enrich the integrated data of PDBe-KB:* The lack of data standards and fragmented nature of specialist data resources is a barrier to the FAIR principle. We will bring together the community experts to define data standards for different types of annotations and integration of these annotations using a community-driven data exchange format will facilitate finding, accessing and reusing sparse annotations in an interoperable manner. Activity I will ensure that PDBe-KB will be an effective platform for the collaborating partners to share and compare their value-added, structure-derived annotations. Furthermore, we will continue to identify gaps in coverage of the various types of annotations in PDBe-KB and bring together community experts to fill in those gaps. The workshops will also facilitate the development and testing of new methods by providing valuable, standardized benchmarking data sets.

*Integration of annotations related to the impacts of disease-associated variants on structure and function:* By bringing together developers of specialist data resources and scientific software tools that can provide information on the effects of amino-acid variation on structure and function, we will establish data standards to represent these data. The data standards will support integration of these annotations in PDBe-KB with particular emphasis on the effect of disease-related mutations on conformational stability. Compiling and integrating these annotations will be highly beneficial when investigating naturally occurring variations, and when used together with all the other types of integrated annotations it may facilitate development of better tools ultimately benefiting the wider biomedical research community.

*Integrating predicted protein structure models (3D-Beacons):* Taking advantage of the highly interconnected data of PDBe-KB will allow the transfer of valuable annotations to structural models based on sequence- or structural similarity when experimentally determined structural data is unavailable. Development of 3D-Beacons infrastructure by incorporating structural models from existing European archives (e.g. SWISS-MODEL ^31^, Genome3D ^40^) and other international resources (e.g. MODBASE ^41^ and Gremlin ^42^) will facilitate an increase in the coverage of structure data in the sequence space. The PDBe-KB data alongside the predicted structure models will potentially allow the scientific community to gain valuable insights regarding the biological context.

The focused activities will take advantage of the existing PDBe-KB infrastructure, and will include developing community-driven data standards and a uniform data access mechanism in addition to novel, portable and distributed web-based visualisation components. Activity I will build on existing collaborations involving five ELIXIR nodes (UK, Italy, Spain, Czech Republic, Belgium).

### Activity II: Open resources for sharing, integrating and benchmarking software tools for modelling the proteome in 3D

***Coordinator: Shoshana Wodak.*** Activity II is about empowering the scientific community to extend the current information on protein 3D structures, protein interactions and assemblies, and extract knowledge from this information by using computational and bioinformatics methods, and integrating biological data from different sources. Its initial focus will be on software tools and benchmark datasets for modeling the 3D structures and conformational flexibility of proteins and protein assemblies, and on community-wide benchmarking activities that advance the field. This focus will subsequently be broadened to include other areas where 3D modelling of proteins and their interactions is most impactful. The following specific aims will be pursued.

***Extend the content of OpenEBench by adding a knowledge base integrating software tools for modeling the 3D structure of proteins, protein complexes and assemblies.*** A variety of software tools are available for modeling protein structures and protein complexes by exploiting available protein sequence data and information on known structures in the Protein Data Bank (PDB). Exploiting these data successfully involves integrating the appropriate set of tools for the problem at hand. The Community will tap into OpenEBench a knowledge portal fostering benchmarking in the life sciences domain and an important component of the ELIXIR Tools Platform, with exemplary datasets of CAMEO and CAPRI already present. The extension will include workflows and guidelines to software tools and servers for modeling protein structures and complexes, based on known structures (templates) in the PDB. More specifically tools for template-based modeling of protein assemblies, and for Integration of template-based modeling of individual subunits, with protein-protein and protein-peptide docking servers developed by members of the CAPRI community. For a more in-depth understanding of the resources please refer to the CAPRI and CAMEO websites. 3D-Bioinfo will build on these efforts and take them to the next level.

***Develop a set of standard freely available tools for evaluating the quality of 3D models of proteins and protein complexes.*** Evaluating the quality and accuracy of 3D models of proteins and protein complexes plays a crucial role in evaluating the performance of molecular modeling methods, and helping methods developers to optimize their procedures. Furthermore, to effectively compare the performance across methods, agreed upon standard quality measures and evaluation protocols are necessary, as implemented for single protein models by CAMEO modeling (3D), the CAMEO Quality Estimation (QE) category and for protein – protein complexes by the CAPRI community. Major goals will include:

1) Full automation of the CAPRI quality assessment procedures for models of protein-protein and protein-peptide complexes, and larger assemblies and making them widely accessible online and for download,

2) Integration of the CAPRI and CAMEO model quality assessment tools,

3) Making the integrated tools available as open software through a community repository (e.g. on GitHub, building on the GitHub established by the CAPRI community).

***Develop a one-stop-shop of benchmark datasets for testing and evaluating methods for generating scoring, and ranking models of protein complexes.*** The availability of appropriate benchmark datasets has been crucial for the development of protein modeling procedures at all levels. Protein docking benchmarks, which assemble high resolution 3D structures of selected sets of known protein complexes and their components ^43, 44, 45^ as well as their experimentally measured affinities ^46, 47^, have been widely used for benchmarking methods for protein-protein docking, and for predicting and scoring protein interaction interfaces. The OpenEBench project within ELIXIR-EXCELERATE (OpenEBench.bsc.es ^48^) has established a transparent data model that allows not only the sharing of benchmarking datasets, but also analyzing and comparing the performance of different prediction algorithms on these datasets.

OpenEBench will thus foster a collection of benchmark datasets relevant to the field of modeling the 3D structure of monomeric, homo-oligomeric and heteromeric protein complexes, extending to proteins - peptide and protein - nucleic acid interactions. It will include: 1) protein docking and affinity benchmark datasets developed by members of the CAPRI community, 2) datasets comprising all the predicted models of protein assemblies submitted to CAPRI and CASP blind prediction challenges, following the examples of the Score-Set ^49^ derived from predicted models submitted to CAPRI, 3) a dynamic benchmark for protein complexes beyond binary interactions, considering different difficulty levels (See DynBench3D; ^50^). In addition a mechanism will be developed for users to contribute datasets ^51^, following well-defined community-approved standards such as the mmCIF based modeling extension developed in collaboration with the RCSB within the macromolecular ModelArchive.org project.

***Develop the infrastructure to manage the CAPRI challenge, in coordination with CAMEO.*** For CAPRI: Develop automated registration and submission procedures (including automated validation of compliance with standard format), as well as tools for accessing and navigating target information, predicted models and prediction results on the CAPRI website. Work on these tasks is currently underway at CAPRI-EBI, but further support is needed to complete it. For CAMEO: CAMEO is currently adding a new category for heteromeric complex modeling. We propose a close collaboration with CAPRI concerning the prediction and scoring applied during fully automated evaluations.

***Develop a knowledge portal to user-friendly bioinformatics and computational tools for modeling conformational flexibility of proteins.*** Adequate modeling of conformational changes is currently a major bottleneck in protein assembly modeling. To foster progress an important first step would be to develop a knowledge portal offering the modeling community at large, workflows and guidelines to various available computational and bioinformatics tools for modeling conformational flexibility. We will use the bio.tools registry established by the ELIXIR Tools Platform as infrastructure for this. bio.tools has tags, one of which is structure prediction, but it should be possible to add more customized tags. With such tools playing an important role in modeling intrinsically unstructured proteins, collaborations with the ELIXIR Community on protein intrinsic disorder on topics of common interest will be undertaken.

### Activity III: Protein-ligand interactions

***Coordinator: Vincent Zoete.*** The biological activity of biomacromolecules is often linked to the three-dimensional recognition and binding of small molecules such as substrates, activators or inhibitors. Indeed, a large fraction of drugs are ligands targeting macromolecules like enzymes, receptors, transporters or ion channels. Consequently, important efforts have been dedicated to develop computer-aided drug design (CADD) and notably structure-based drug design (SBDD) approaches over the last decades, contributing significantly to the design of small molecules of therapeutic interest.

The field of drug discovery and development will face several challenges in the future. The on-going needs in medicinal chemistry prompts a dramatic demand for new molecular entities and the exploitation of chemical spaces that are yet unexplored. Despite important progress over the last few decades, toxicity issues remain a problem for small compounds. This has to be addressed through increased specificity, but also via a better characterization of the possible targets and the anticipation of multiple off-target effects. Toxicogenomics, pharmacogenomics, and phenotypic screening data should be collected, organized and disseminated to get a clearer overview of biomacromolecule-ligand interactions, and to ultimately predict *in silico* the poly-pharmacology of the compounds. New target classes are also emerging, beyond the usual well-defined binding pockets, including among others the interaction of proteins with other proteins, nucleic acids, lipids or sugars. These additions in the target classes are mirrored by the use of new classes of ligands, including peptides and macrocyclic compounds. These new types of target-ligand interactions will foster the development of novel *in silico* approaches, which will require new algorithms and thorough evaluations of their descriptive and predictive capacities.

Among other *in silico* technologies, structure based drug design (SBDD) methods remain in great need of comprehensive evaluations of their performance and domain of applicability. In particular, there is a need for improved docking and scoring methods. Although protein-ligand complexes can be predicted for small ligands and almost rigid proteins, large, flexible molecules like peptides or macrocycles and proteins with flexible binding regions are still very difficult to handle. The reliable prediction of the binding free energy of a protein-ligand complex also remains a major challenge. Better methods making use of precise chemical models, modern optimization techniques and innovative scoring approaches are still much needed but must be accompanied by comprehensive and rigorous evaluations of their performance and domain of applicability. This should go hand in hand with the creation of processing pipelines for the proper use of structural data, and of standardizing tools for IO.

The efficiency of SBDD tools often depends on molecular/physicochemical properties such as the charge, polarity or size of the protein binding site and ligand, as well as on the target class of the biomacromolecule or the chemical class of the ligand. To address this, benchmark sets should clearly list and quantify these different properties for each complex with suitable descriptors, transparent for the user community. For Activity III, we plan the following goals:

***Creation of benchmark datasets to assess structure-based drug design tools.*** Benchmarking studies performed so far, including those carried out in the CSAR ^52^ or D3R Grand Challenge ^53^ which focus on compound series consistently measured within one lab/institute - rely on datasets of limited size and diversity, precluding large-scale, FAIR comparisons of their performance, especially as a function of ligand and binding site properties. Activity III will therefore involve:

1) Building benchmark datasets, extracted from the PDB and curated, for assessing SBDD tools on a large-scale, under well-defined FAIR conditions, thereby complementing efforts such as the D3R grand challenges. A particular effort will be devoted to collecting complexes involving peptides and macromolecules.

2) Quantifying different properties (e.g. charge, polarity, size, flexibility of the binding site and ligand etc.) for each entry in the benchmark, to enable evaluation of SBDD tools as a function of these properties.

3) Developing links to other databases and standardizing the retrieved data to complement the information provided for each protein-ligand complex in the benchmark sets; notably, collecting information on ligands in collaboration with PDBe-KB, and associating complexes with reliable binding affinity data using databases like ChEMBL ^54^ and Pubchem ^55^.

4) Adding information on the non-bioactive conformations of ligands to standardize the comparison of docking calculations starting from such geometries.

5) Adding information on experimentally determined non-active compounds (taken e.g. from ChEMBL ^54^) to be used as negative examples for testing virtual screening procedures.

***Dissemination and promotion of the benchmark datasets and results.*** Benchmark datasets will be made publicly available by following Open Access and FAIR principles via a collaboration with the OpenEBench project within ELIXIR EXCELERATE. The latter will also allow the sharing of benchmarking results, and comparing the performance of different prediction algorithms under FAIR conditions. Preferred standardized benchmark workflows and protocols will be published, to guarantee an objective comparison of different tools. Researchers will be encouraged to evaluate their preferred sets of SBDD approaches using the above-mentioned standardized benchmarking workflows and protocols, and to report their results. We will collect the latter and provide them to the community. This activity will be of major value to Pharma and Biotech researchers, who acknowledge the importance of standard benchmarking protocols.

***Biomacromolecule-ligand interactions for every scientist, educational aspects.*** Despite significant interest in using modelling approaches among life scientists, the use of biomacromolecules structures in molecular design largely remains a domain for experts. However, complex data preparation and association could be largely automated resulting in easier-to-use software and substantially lowering the usage barrier for life scientists. By encouraging the development of tutorials guiding the application of modelling and the interpretation of the achieved results, we will endeavour to open the world of structure-based modelling to the broader life science community.

### Activity IV: Tools to describe, analyse, annotate, and predict nucleic acid structures

***Coordinator: Bohdan Schneider.*** The ultimate goal and vision of Activity IV is to encourage development and use of software tools to describe, analyse, annotate, and predict nucleic acid (NA) structures. The availability and sophistication of tools dealing with various hierarchies of the nucleic acid structure lag behind the tools used to explore protein structures and this situation must be remedied. In particular, standards are needed for initial model building and refinement of nucleic acid molecular structures. This task has become urgent as new techniques, including, but not limited to cryo-EM, are generating experimental data on 3D structures containing RNA and DNA molecules, such as ribosomes, spliceosomes, polymerase assemblies, and histone complexes, faster than ever before. The modelling of large nucleic acid molecules into low-resolution electron densities is particularly challenging. The RNA structural bioinformatics community has provided prototype tools, with which to model RNA and RNA-protein complexes based on experimental data, but these tools are currently not compatible with community-wide standards describing RNA and DNA conformational space and geometry at the local (nucleotide or dinucleotide) levels.

Therefore, efforts need to be directed towards formulating community-accepted benchmarks that integrate the different levels of nucleic acid structure descriptions, and more generally to improving software tools that describe, analyse, annotate, and model nucleic acid structures. To enable these developments, Activity IV will focus on the following specific goals:

1) Cataloguing software tools for building nucleic acid models based on their sequences alone as well as for modelling their 3D structures using experimental data, and facilitate integration of these tools.

2) Coordinating the unification of the existing NA geometry standards and formulate specifications for missing standards.

3) Developing benchmarks dataset for evaluating the quality of predicted or experimentally determined NA structures.

To limit the redundancy and increase the synergy between the methods, databases, web services, and other tools, we plan to continuously update the catalogue of software tools developed by the RNA tools and software consortium, extend these tools to DNA structures and enable integration of emerging tools. These efforts will build on existing ontologies while preserving consistency with new extensions.

This integration effort will require a significant level of interoperability between data exchange protocols and software, which will be implemented following FAIR principles. Dealing with software to solve, model, and refine NA structures based on the experimental data will require a close collaboration with experimentalists. Hence joining forces with Instruct-ERIC will be essential, for reaching all the stated goals, including the development of benchmarking tools and standards.

We list a few examples of steps, which could assist useful integration of the existing tools:

1) Adding links from existing servers to other tools, especially those linking 2D and 3D structure prediction. The RNA Tools and Software Consortium have already recapitulated methods for RNA secondary structure prediction and to some extent RNA 3D tools. The RNA Puzzles community of RNA structural bioinformaticians ^16, 17, 18^ has developed a set of tools for linking 2D and 3D structures, software for RNA 3D structure model evaluation also exists, e.g. RASP ^56^ or MacroMoleculeBuilder, MMB ^57^.

2) Unifying the libraries of RNA/DNA dinucleotide fragments based on the analysis of experimental structures ^58, 59^ and trinucleotide fragments ^60^. Ultimately, the community should reach a consensus on what is the meaning of “preferred”, “allowed” and “wrong” conformers in analogy with the use of these terms in the Ramachandran plot.

3) Integrating and benchmarking methods dealing with RNA structures, e.g. SimRNA ^61^, RNAComposer ^62^ MMB ^57^, FARNA/FARFAR ^63^, Web-Beagle ^64^.

4) Strengthening the collaboration with developers of the main experimental nucleic acid structure determination software tools (e.g. REFMAC ^65^, COOT ^66^, Phenix ^67^, PDB-REDO ^68^) to encourage consistent handling of NAs.

The goals of Activity IV are quite ambitious, and their implementation will require close and friendly collaboration of all research teams willing to participate; including the teams involved in this Activity, and other teams active in the field. The teams not involved in Activity IV will be encouraged to join. Successful completion of the stated goals will also depend on the close collaboration of scientists grouped under other infrastructure projects in Europe and beyond. New tools, standards and benchmarks developed for NA validation will be communicated to the experimental structural biology community, mainly Instruct-ERIC but also EuroBioImaging, to ensure consistency across the different research communities.

As a part of the 3D-Bioinfo Community, we plan to hold regular meetings and workshops and web conferences, to informally discuss progress and help identify problems that can be addressed collectively. Unlike other 3D-Bioinfo Activities, Activity IV involves a relatively new community that is less well established and will require organising workshops to encourage collaboration and to enable updating of tool catalogues and ontologies. As with the other 3D-Bioinfo Activities, we will coordinate organisation of the workshops with the ELIXIR’s training portal TeSS where appropriate. The first workshop of the Activity IV is going to take place in May 2020.

### Future 3D-Bioinfo activities

The above mentioned themes will not only form the initial focus for 3D-Bioinfo but the steering committee will actively monitor the emergence of new technologies and/or new research fields relevant for bioinformatics approaches, which then can be fostered further as new activities. For example, in the field of protein design the overarching aim is to enable completely rational design of proteins with customized biological functions e.g. novel biocatalysts for Green Chemistry to meet sustainability and environmental challenges. In order to foster new developments focused courses/workshops will be organised on such topics. For example, a course or workshop on using biocomputing to understand and engineer biocatalysis is being planned. 3D-Bioinfo will continuously seek to integrate biocomputational efforts with experimental studies in order to systematically generate, test and critically assess new hypotheses on the fundamental properties of highly active enzymes and binding proteins. This would very much increase our quantitative understanding and enhance the capabilities for the rapid generation of binders, inhibitors and biocatalysts for a range of applications in research, technology and medicine.

## Interaction of 3D-Bioinfo with other research communities

### Alignment with the ELIXIR platforms

All the above 3D-Bioinfo Activities will engage with the ELIXIR platforms as described below.

*Interoperability platform* – The outcomes of our projects must be easy to discover, to access and to integrate into users’ pipelines. This necessitates the use of standardised file formats, metadata, vocabularies and identifiers. We plan to include our resources in FAIRshairing and Identifiers.org. For all Activities we will organise community workshops to develop data exchange and retrieval standards to improve compliance with FAIR principles. Participating teams will also adopt BioSchemas ^69^, a European led initiative. For example, different community-wide standards for evaluating predicted models of proteins and protein complexes would be integrated to promote community-wide use. FAIR-ification of benchmark datasets will be undertaken. To implement the 3D-Beacons infrastructure, we will implement a common API specification for macromolecular structure data from both experimentally determined and predicted models. Where possible, the services and tools will also be linked via workflows using common workflow language to help with interoperable workflow software development.

*Data platform* – We will link key structural bioinformatics data resources to drive the use and re-use of data. For example, data from five UK based structure prediction resources have already been integrated via Genome3D, and ELIXIR implementation studies are already supporting further integration of the data in Genome3D with data from SWISS-MODEL, developed by the Swiss node. In addition, Activity I will be responsible for the integration of data on known and predicted functional sites, from a large number of participating European groups, in PDBe-KB. Information on the structural impacts of genetic variations predicted by multiple groups will also be integrated and novel visualization strategies for presenting this integrated data will be developed. These will need to clearly distinguish between experimentally known and predicted data. Activity II will be responsible for integrating various benchmark datasets on predicted protein complexes and assemblies, and on experimentally determined complexes annotated with data from other sources (in close coordination with Activity I). Activity III will also integrate benchmark datasets. For example, we will establish Open Access benchmarks sets under FAIR principles, for assessing structure-based drug design applications. This must be done in a robust, sustainable and scalable data ecosystem, allowing the use and re-use of the data, in line with the ELIXIR Data Platform goals.

*Tools platform* – The participating tools and resources will be registered in BioTools to make them discoverable and sustainable. Currently, there are several hundred tools relating to structural bioinformatics registered in BioTools covering a range of themes. Extending the repertoire of structural bioinformatics tools in BioTools will a) ensure reproducibility, b) allow scaling up by working in cloud environments, e.g. EOSC c) make tools widely available and sustainable for non-expert users. Containerization of these tools in BioContainers will support development of complex workflows. We will use the benchmarking infrastructure OpenEBench to assess tools and help methods development. For example, all tools related to Activity I will be registered in BioTools and the developers will have the opportunity to access expertise on containerization; for Activity II, these will incorporate exemplary datasets from CAMEO and CAPRI. Workflows for modeling protein conformational flexibility and for modelling protein assemblies in the context of Cryo-EM structure determination will be designed in collaboration with respectively, BioExcel and Instruct-ERIC. The development of improved tools for validation of nucleic acid models in Activity IV feeds directly into this platform and all new methods will be registered with BioTools.

*Compute platform* – The ELIXIR Authentication & Authorisation Infrastructure (ELIXIR-AAI) which connects to other European AAI initiatives like EGI-CheckIn, INSTRACT ARIA, will be adopted to support depositions. We will link with the compute platform activities to address scalability of the underlying infrastructure for data transfer as well as data access. For example, access to the ELIXIR compute infrastructure and to the European Open Science Cloud resources (EOSC-Hub) will be explored, to enable large-scale assessment of predicted models of protein assemblies in CAPRI prediction rounds, and to disseminate software tools and web-services. Similarly, groups providing large-scale protein structure predictions and variant impacts will seek to benefit from access to the ELIXIR compute infrastructure. We will adopt ELIXIR Authentication & Authorisation Infrastructure (ELIXIR-AAI) to support deposition of ligand-protein complexes or benchmark results into our future infrastructure. We also need to provide an easy way to store and synchronise our datasets and users’ benchmarks results across ELIXIR and other e-Infrastructures.

**Figure 4.**
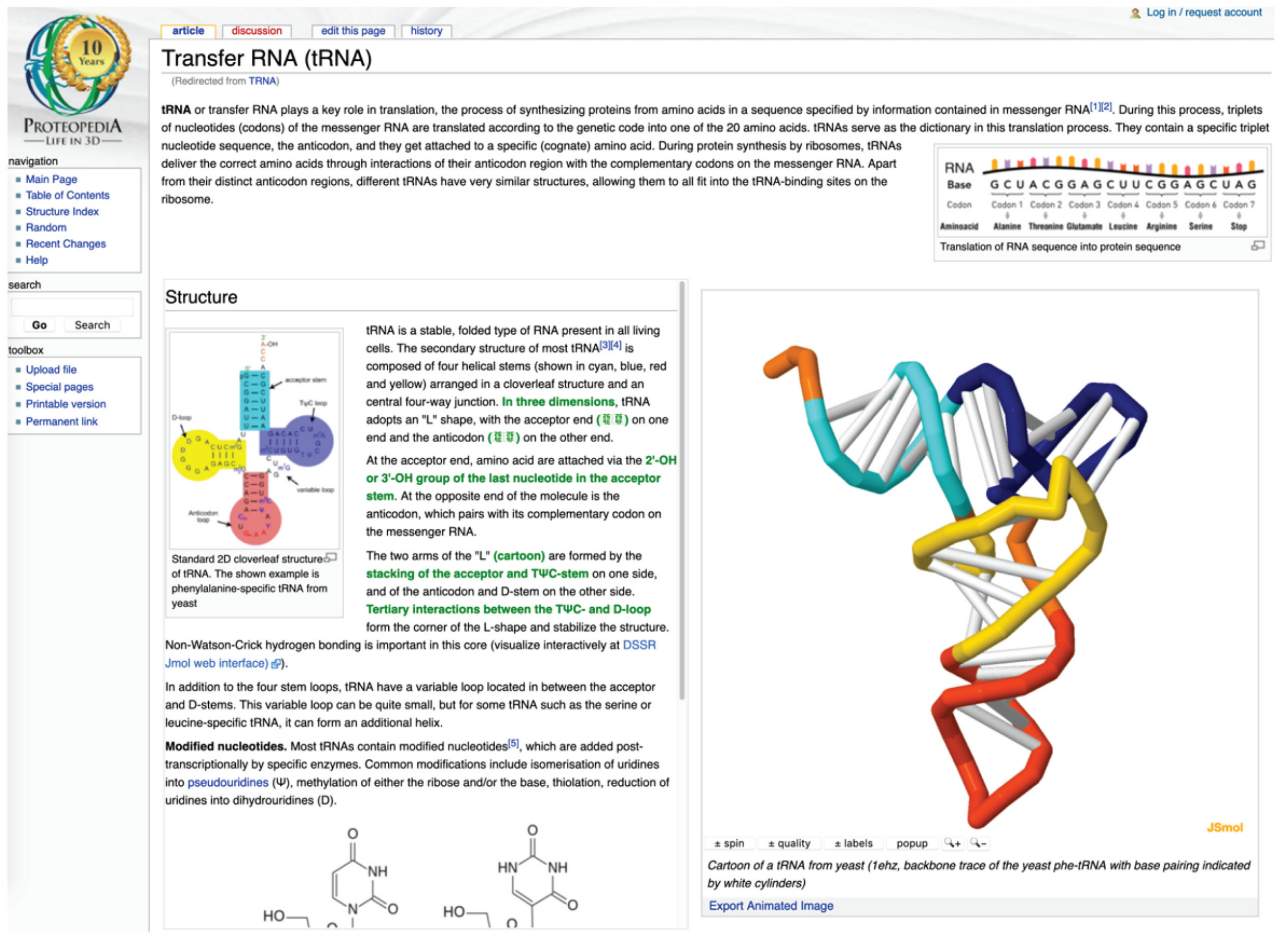
The tRNA page from Proteopedia [ http://proteopedia.org/w/TRNA]. tRNA plays a key role in translation, the process of synthesizing proteins from amino acids. The two arms of the "L" shaped molecule (cartoon) are formed by the stacking of the acceptor and TΨC-stem.

*Training platform* – PDBe-KB and participating data resources will work to add training workflows to the TeSS portal. As mentioned already, ELIXIR-UK funding has already supported preliminary work on these workflows involving collaborations between multiple partners. Proteopedia, which is being developed in collaboration between the Israeli node and PDBe at the hub, will also be a valuable mechanism for training and outreach (see Figure 4). Connections with existing initiatives such as the BioExcel Knowledge Resource Center will be established. The BioExcel knowledge resource center is a repository for computational biomolecular training resources. The resources are primarily online based, such as tutorials, online courses and videos but also include face-to-face events.

With regards to protein ligand interactions, we will train researchers on the best way to retrieve and use our benchmark datasets, and promote the usage of preferred benchmark conditions. The Molecular Modeling Group of the SIB Swiss Institute of Bioinformatics will enhance the Drug Design Workshop. This is a web-based educational tool ^70^ to introduce structure-based computer-aided drug design to the general public. This online workshop constitutes a helpful tool to introduce the concepts of structure-based drug design to young students (15−19 years old) and to the general public. It can also be used as an introductory tool for more advanced students. Several routes of enhancements will be followed, including a better visualization and analysis of the ligand-protein complex, or the selection of new protein targets.

Building on the experience of the CAPRI community in organizing the well- attended EMBO courses on Integrative of Biomolecular Complexes (3 editions so far), Activity II will coordinate various training activities and workshops on teaching non-experts how to use protein structure and assembly prediction tools and how to benchmark new prediction methods using various datasets assembled in Activity II. Training workflows will be derived and added to the TeSS portal. Activity II will also help coordinate training tasks related to protein structure prediction in the Meet-U initiative. This is an innovative pedagogical initiative created in 2016 that teaches MSc students by involving them in real world research projects, with results evaluated by experts in the field during a final scientific symposium. Meet-U is currently implemented as a collaborative course between three universities of Paris/France area: Sorbonne Université (SU/UPMC), Universités Paris-Sud/Paris-Saclay, and Université Paris-Diderot. It is proposed to implement an international version of Meet-U, involving universities linked to ELIXIR nodes across Europe.

There has also been some support from the ELIXIR UK node to develop training workflows in protein structure prediction and variant impact analysis. This has enabled pilot work and the establishment of small-scale training workflows in the ELIXIR TESS registry. For all activities, we will organise training workshops for trainers and users, and add workflows to TeSS where appropriate.

### Connection to other communities and European infrastructure initiatives

3D-Bioinfo community users: The 3D-Bioinfo community bridges several infrastructures and their providers as well as users, namely people and tools of structural biology (Instruct-ERIC, iNEXT), cheminformatics (OpenScreen), system biology (ISBE), molecular simulations (BioExcel) and the proposed community for intrinsically disordered proteins (IDP). In addition, the value of structural data in providing insights into the impacts of genetic variations has led to involvement of some of the 3D-BioInfo participating groups with the ELIXIR Rare Disease Community. Similarly, work in all Activities on structural impacts of residue mutations and other research fields of protein engineering and nucleic acid analogues would clearly enable links with the newly emerging ELIXIR Community on Synthetic Biology. The action plan of this community includes the development of new strains with designed metabolic pathways and expression systems. The synergy of the expertise in the 3D-Bioinfo and Synthetic Biology communities will therefore be very important to implement the new biotech applications that can be expected to be generated through the acquired knowledge and expertise generated by the 3D-Bioinfo ELIXIR community.

The tools and services offered by the proposed 3D-Bioinfo Community already have many millions of users per year and the new Activities, described above, will undoubtedly broaden this scope.

As regards potential overlap with Instruct-ERIC, the 3D-Bioinfo community develops tools that go beyond the scope of structural biology and are not covered by the Instruct-ERIC initiatives and efforts. We will work closely with Instruct-ERIC on common areas of interest (e.g. methods for validating experimental protein and nucleic acid structures) and seek support for joint implementations studies.

### Connection with industry

Industry and SME involvement – As mentioned already, Activity III (linked to structure-based drug design) is of particular relevance for the pharmaceutical and biotechnology industries, particularly those using computer-aided structure-based drug design programs. Companies involved in the development of scientific software as well as novel bioactive compounds acknowledge the importance of developing benchmarking protocols, and participate in the D3R Grand Challenges by providing undisclosed data. Furthermore, as regards Activity I, PDBe-KB has interactions with OpenTargets platform and provides annotations to major data resources (UniProt, InterPro and Pfam). Development of data standards and distributable infrastructure would further improve accessibility of the added value annotation data for industry users where all four Activities will contribute. We also expect to build up links to the pharmaceutical Industry via the collaborations with BioExcel, and Instruct-ERIC. Future 3D-Bioinfo Activities around protein and enzyme engineering are expected to provide strong links with the industry and it is anticipated that these links will very much strengthen the competitiveness of the developing European biotechnology SMEs.

### Integration at a global level

Clearly the ontologies and data exchange formats established and endorsed through 3D-Bioinfo enabled collaborations will be valuable in a global context as well as across Europe. In fact many of the initiatives we will foster such as PDBe-KB, CAPRI and CAMEO already involve other international partners outside Europe. Furthermore, 3D-Bioinfo groups are involved in organising the international community-wide CASP benchmarking of protein structure prediction. We will support and where possible engage in initiatives for global health, for example the Global Alliance for Genomics and Health (GA4GH). We plan to present activities and output from 3D-Bioinfo in special sessions or technology tracks at the European ECCB and international ISMB conferences, the latter of which is held in Europe on alternate years. This will publicise our activities to the wider bioinformatics community, enable us to recruit additional European participants and promote links with other international initiatives.

## Conclusions

This proposal capitalises on the extensive European structural bioinformatics expertise. It provides a discussion and action framework for joint development of current and future activities within the European structural bioinformatics community. 3D-Bioinfo will very much foster the interactions with experimental research groups to efficiently reach a better understanding of proteins and their functional properties at a quantitative level. 3D-Bioinfo will also foster focused outreach and training activities. The outlined aims of our planned Activities should facilitate valuable coordination for optimal use of resources and exploit ELIXIRs platforms to good purpose. As demonstrated in our background to this paper and the many tools/resources listed in the BioTools, Europe is very strong in this area of research and the establishment of the 3D-Bioinfo Community will assist in promoting research interactions, integration of data and tools and standardised practices, making it even stronger. Furthermore, the Community will facilitate the translation of structurally derived insights - in medicine (pharmaceuticals, diagnostics), agriculture, and sustainable production methods. Finally, 3D-Bioinfo will facilitate collaborations worldwide and promote European research in structural bioinformatics, on a global scale.

## Data availability

### Underlying data

No data are associated with this article.
