# Supplementary material for: A community proposal to integrate structural bioinformatics activities in ELIXIR (3D-Bioinfo Community)
Source: F1000Res. 2020 Apr 22;9:ELIXIR-278. [Version 1] doi: 10.12688/f1000research.20559.1 (PMC7284151; doi:10.12688/f1000research.20559.1)
Supplement: Peer review history

# Peer review: Reviewer response for version 1

**DOI:** 10.5256/f1000research.22602.r63105

**Published:** 2020-06-11

**Authors:** Andras Fiser

**Affiliations:** 1 Department of Systems and Computational Biology, Albert Einstein College of Medicine, The Bronx, NY, USA

The manuscript of Orengo et al presents the goals of the Elixir initiative in the context of historical achievements in the field of 3D Bioinformatics.  It is a conceptual piece to provide guidance to an entire field and therefore I would reflect on that.

 ‘Computational approaches in biology’ always suffered from an identity problem and I believe this manuscript does not help to resolve it and as such it might be a missed opportunity. One major issue is if one approaches computational structural biology as a discipline or a technology. I believe Elixir efforts all about boosting the potential of this discipline but the scope of this effort is still presented rather as a technology that can support other disciplines.  

 The first sentence of the abstract immediately leaves this question un-answered “Structural bioinformatics provides the scientific methods and tools to analyse, archive, validate, and present the biomolecular structure data generated by the structural biology community.”. By leaving out aspects of “modeling” from the possible contribution, which I believe is a frequent and important aspect of computational structural biology, the authors restrict the activities for retrospective analysis of experimental data, to “bioinformatics”, i.e to “organize, analyze an manipulate data”.  And do not make the argument that “modeling”, i.e. gaining new, testable hypothesis and generating new knowledge is also part of this field (e.g. structural modeling of proteins without experimental structures; docking receptors and ligands in so far unseen complexes; predicting new receptor ligand interactions; predicting  and modeling protein drug interactions; modeling /simulating protein motions;  modeling enzyme kinetics with molecular dynamics or quantum mechanical simulations; etc.).

 NIH made an attempt to distinguish between “bioinformatics” and “computational biology”. If I try paraphrase it in a 3D context, 3D-Bioinfirmatics is: research, development, or application of computational tools and approaches for expanding the use of 3D biological data, including those to acquire, store, organize, archive, analyze, or visualize such data. In contrast to 3D-Computational Biology of biomolecular structures, which is the development and application of data-analytical and theoretical methods, mathematical modeling and computational simulation techniques to the study of macromolecular structures.   

 This review so far was concerned with the first sentence of the Abstract only. The rest of the paper does touch on a number of innovative, activities that generate new knowledge, i.e. a discipline like activity, but at least to this reviewer this is not formulated in the framework enough.

Is the topic of the opinion article discussed accurately in the context of the current literature?

Yes

Are arguments sufficiently supported by evidence from the published literature?

Yes

Are all factual statements correct and adequately supported by citations?

Yes

Are the conclusions drawn balanced and justified on the basis of the presented arguments?

Yes

Reviewer Expertise:

computational structural biology

I confirm that I have read this submission and believe that I have an appropriate level of expertise to confirm that it is of an acceptable scientific standard, however I have significant reservations, as outlined above.

# Peer review: Reviewer response for version 1

**DOI:** 10.5256/f1000research.22602.r63141

**Published:** 2020-06-09

**Authors:** Roland L. Dunbrack

**Affiliations:** 1 Fox Chase Cancer Center, Philadelphia, PA, USA

This opinion article summarizes the activities, both existing and planned, of the 3D-Bioinfo Community within the ELIXIR biological data infrastructure project in Europe. As such, it represents a useful statement of purpose that can be used to guide the efforts of the many participating groups and in the future as a potential means of evaluating the success of those efforts.

 The first section of this article (“Major European contributions in structural bioinformatics”) describes the historical impact of European research groups in the field of structural bioinformatics. These contributions are extensive and indisputable but I am not sure that most scientists will find it very interesting. I suppose it is necessary for European funding bodies to hear it, and in the United States we often find ourselves doing the same thing while trying to secure funding for bioinformatics infrastructure like the PDB from the National Institutes of Health and the National Science Foundation. Perhaps this section could be shortened, or it could briefly mention parallel developments in other parts of the world. For instance, Helen Berman’s efforts in transforming and remediating the PDB into a modern bioinformatics infrastructure resource at Rutgers University starting in 1998 and the establishment of the wwPDB in 2003 with PDBe and PDBj deserve some comment.

 The second section briefly describes “Major challenges that 3D-Bioinfo will help to address” including proteome-wide protein structure prediction, establishing benchmarks and confidence assessments for structure prediction accuracy, evaluating the impact of sequence variants on protein function and disease, improvements in structure determination by cryo-EM, SAXS, integrative modeling, and other methods that depend on the appropriate use of high-resolution structural data from crystallography, and the prediction of protein-ligand interactions. All of these require significant infrastructure and integration and interoperability of tools and data, and 3D-Bioinfo is well placed to operate in this sphere. All of these make sense, even if they are fairly obvious.

 It may be an unpopular opinion, but I don’t think protein structure prediction on the proteome scale is such a worthwhile goal, except for organisms that are of significant experimental interest. Because we can make models of millions of proteins from 100s or 1000s of organisms, doesn’t mean we should. Most of that data would be unused. The fact that it “remains computationally expensive to build 3D models on a proteome-wide scale” as the article states is not really an issue. The more important issue is making biologically relevant models of proteins – as homo- and heterooligomeric complexes, interacting with ligands and nucleic acids, and in different functional states (e.g., the active and several inactive conformations of kinases).

 One challenge that was left out is the validation of experimental structures at the atom/residue level (amino acids, nucleic acid bases, and ligands), and more importantly, their re-refinement with new methods as they are developed. I am a fan of two tools developed by some of the authors on this paper, EDIA for evaluating electron density in X-ray crystal structures on an atom-by-atom basis, and the PDB-REDO database of re-refined crystal structures.

 The next section is a general statement of the goals of 3D-Bioinfo: 1) improved interoperable data standards 2) planning for sustainability and integration of data resources; 3) standards, benchmarking, and validation of prediction tools; 4) access to high compute facilities; 5) integration of structural data with other biological data and databases; and 6) training and outreach in bioinformatics. These are all very worthy, if rather broad and general goals.

 The next section on “Links between 3D-Bioinfo and the wider European research environment” reads more like a grant progress report and is probably of little interest to the general reader, especially the section on the launch meeting, which could be deleted. This section mentions collaboration with the Protein Structure Initiative (PSI) by some of the current 3D-Bioinfo participants, but the PSI was disbanded in 2015 and this reference should be removed. This section mentions Instruct-ERIC, and it would be helpful to describe what are the similarities and differences in the goals of 3D-Bioinfo and Instruct-ERIC, which may not be that familiar to non-European structural biologists. Instruct-ERIC is more focused on experimental technologies.

 Finally, we get to the meat of the matter – the four “Activity” areas: I. Infrastructure for FAIR structural and functional annotations; II. Open resources for sharing, integrating and benchmarking software tools for modelling the proteome in 3D; III. Protein-ligand interactions; IV: Tools to describe, analyze, annotate, and predict nucleic acid structures. (Full disclosure: I am a participant in Activity II).

 The first activity (I) is to improve annotations of protein structures, in particular to bring 3rd party annotation resources into the PDBe-KB (knowledge-base) via a standard data exchange format (e.g., json-based formats). These will include functional site annotations, predicted structures, and impact of disease-associated variants on structure. This is appealing to me, since my group generates these kinds of annotations, for example on antibodies, kinases, and protein-protein interactions. Access to these annotations via the PDB would greatly increase the impact of such resources and the depth of annotation of PDB structures. One example that is already in practice is the import of QSBIO and 3Dcomplex annotations for protein assemblies of X-ray structures in the PDB.

 Activity II is focused on integrating and benchmarking software for modeling proteins in 3D, including in particular modeling or annotating protein assemblies and conformational flexibility. This aspect of 3D-Bioinfo will include integration of CAMEO and CAPRI, both of which assess structure predictions of proteins or protein complexes, into OpenEBench, which is also part of ELIXIR. It will also involve developing benchmarks for the prediction of the structures of protein assemblies (e.g. for benchmarking docking calculations) and providing a portal for community-derived benchmarks. While there are many such benchmarks, they are not fully integrated into one resource which will likely be productive in the way that CAMEO, CAPRI, and CASP have been. This section could use a more succinct statement of what is missing in existing benchmarks and what 3D-Bioinfo would do to fill the gaps.

 Activity III is focused on developing and disseminating benchmarks  for modeling protein-ligand complexes, including peptides and macrocyclic compounds, and their affinities. The inclusion of experimentally determined non-active compounds (for the construction of negative data sets) is an important suggestion in the paper. Another goal is to “open the world of structure-based modeling to the broader science community.” Worthy, but easier said than done, and it is not clear what this would look like exactly.

 Activity IV is focused on refinement of experimental structures, structural bioinformatics, structure prediction, and benchmarking studies of nucleic acid structures, which, as the authors point out, lag behind comparable efforts on proteins. As with the other Activities, the efforts will  be on establishing benchmarks, collecting software tools, and enhancing interoperability. The authors explicitly propose bringing additional research groups outside of 3D-Bioinfo into Activity IV, presumably including those outside of Europe, which would greatly strengthen all of the activities of 3D-Bioinfo.

 The paper concludes with a discussion of further interactions of 3D-Bioinfo with other research communities including: (1) ELIXIR “platforms” on Interoperability, Data, Tools, Compute, and Training; (2) European bioinformatics infrastructure initiatives such as those on cheminformatics, systems biology, and intrinsically disordered proteins; (3) connections with industry; and (4) global initiatives. The last of these is given rather short shrift, considering that the efforts of a European 3D-Bioinfo project will not succeed if they don’t include input and adoption by research groups worldwide. In particular, it is surprising not to see any discussion of interactions with the RCSB or PDBj, which are after all components of the World-Wide PDB (wwPDB), along with PDBe.

 Very minor:

1. Figure 2: typo, “Uniport” --> “Uniprot”
2. p. 14: “EMBO courses on Integrative of Biomolecular Complexes” --> “EMBO courses on Integrative Modeling of Biomolecular Complexes”

Is the topic of the opinion article discussed accurately in the context of the current literature?

Yes

Are arguments sufficiently supported by evidence from the published literature?

Yes

Are all factual statements correct and adequately supported by citations?

Yes

Are the conclusions drawn balanced and justified on the basis of the presented arguments?

Yes

Reviewer Expertise:

Structural bioinformatics and protein structure prediction

I confirm that I have read this submission and believe that I have an appropriate level of expertise to confirm that it is of an acceptable scientific standard, however I have significant reservations, as outlined above.

# Peer review: Reviewer response for version 1

**DOI:** 10.5256/f1000research.22602.r62621

**Published:** 2020-05-22

**Authors:** Silvio Tosatto

**Affiliations:** 1 Department of Biomedical Sciences, BioComputing Laboratory, Padova, I-35121, Italy

The manuscript by Orengo et al. describes the proposed activities of the ELIXIR 3D-Bioinfo user community. This is a useful and necessary contribution to describe the scope and plans of a new ELIXIR user community. The introduction provides a recapitulation of the uses and relevance of structural bioinformatics in different contexts. As the authors note, this is a mature field where several key data resources and initiatives alredy exist. The manuscript reads well and tries to make convincing arguments for the proposed activities.

**Specific comments:**

*From the scientific point of view:*

1. Limits of structural data. Structural data is very important for many purposes, and the authors make this point clearly. However, it has also limits which should be spelled out clearly to avoid the impression of being a panacea. One limit is the availability of experimental data, with some molecules defying structural characterization. A more specific limit is also related to intrinsically disordered proteins (IDPs). This is a different (sub-)field which is not covered by 3D-Bioinfo and has spawned a separate ELIXIR user community with different priorities dictated by the lack of structural data.
2. Conformational variability. This important aspect is mentioned several times and forms a continuum between fully rigid proteins on one end and IDPs at the other. It is important to clarify this concept in the manuscript. By doing so it also offers areas for further collaboration in ELIXIR across user communities.
3. Proteomics Standards Initiative (PSI). Part of the Human Proteomics Organization (HUPO), it should be referred to as HUPO-PSI. This is the main standardization body in the field and an obvious choice for promoting the standards 3D-Bioinfo aims to establish. As such, it should be introduced in the manuscript and its implications discussed. Of note, at present there is no HUPO-PSI working group in the areas covered by 3D-Bioinfo.
4. Activity 1. The PDBe-KB part is well described and convincing. There is clear potential for synergies on representing IDP data.
5. Activity 2. The dscription appears somewhat repetitive and its focus may be improved. OpenEBench is a good idea for CAMEO and CARPI, but how can integration be optimally achieved? The comformational modeling portal part is also a bit unclear, especially in light of the overlap with IDPs. A suggestion would be to highlight this as a way to foster collaboration with the IDP user community.
6. Activity 3. The relevance of protein-ligand interactions is out of question. Since this field is of such pharmaceutical relevance, it is also quite mature. It is however not immediately clear what novel benefit 3D-Bioinfo can provide in terms of instrastructure beyond coordination.
7. Activity 4. This is scientifically perhaps the most "novel" part of the proposed activities, as nucleic acid structures have traditionally been a minority in the field. The description seems more typical of a COST Action setup though and the link to ELIXIR should be improved. E.g. how can the ELIXIR services help to achieve the goals of this activity?
8. Future 3D-Bioinfo activities. This section appears somewhat redundant. In the Green Chemistry example it is unclear how this relates to ELIXIR.
9. ISCB. Given the prominent role of at least one main author in the ISCB leadership, this reviewer was expecting a stronger commitment regarding synergies with the society and its COSIs (most notably 3D-SIG). The goals clearly overlap and can benefit from each other.

*From the ELIXIR point of view:*

1. Platforms. The description of interactions with ELIXIR platforms appears to denote a somewhat cursory knowledge of their activities. The interaction with OpenEBench is well described and convincing. The Data platform description is good for PBDe-KB but after this does not appear related to platform activities. The Compute platform description is also unclear. In all cases, the question is: how will 3D-Bioinfo make convincing use of platform services?
2. Other user communities. A more thorough description of synergies with existing ELIXIR user communites would be welcome. This should also be separated from non-ELIXIR initiatives (e.g. Instruct, iNEXT, etc.). Of note, the IDP user community is already established and not proposed. Likewise, "Synthetic Biology" should probably be Microbial Biotechnology. The Proteomics community is an easy connection but missing. Other communities, e.g. Plants, may also be relevant. In general, it would be good to have specific items for collaboration listed.

**Minor points:**

- The manuscript is somewhat redundant and a bit lengthy at 16 pages without references. Streamlining it would improve focus and can contribute to mitigate some of the concerns above.
- The text refers extensively to "Europe" and "European" resources, ca. 60 times throughout. While this is a truism for ELIXIR, it sometimes feels just a bit too much emphasis in its present form.
- Figure 1 contains data for the PDB up to the year 2016 (a), 2017 (b) and 2018 (c). It should be updated and unified with data until 2019.
- When listing COST Actions (p. 8, left column, bottom), a unified format should be used. I.e. either list all titles or none.

Is the topic of the opinion article discussed accurately in the context of the current literature?

Yes

Are arguments sufficiently supported by evidence from the published literature?

Yes

Are all factual statements correct and adequately supported by citations?

Yes

Are the conclusions drawn balanced and justified on the basis of the presented arguments?

Partly

Reviewer Expertise:

bioinformatics, computational biology, structural bioinformatics, intrinsically disordered proteins, databases, tools, infrastructure

I confirm that I have read this submission and believe that I have an appropriate level of expertise to confirm that it is of an acceptable scientific standard, however I have significant reservations, as outlined above.

# Peer review: Reviewer response for version 1

**DOI:** 10.5256/f1000research.22602.r63104

**Published:** 2020-05-21

**Authors:** Isaure Chauvot de Beauchene, Sjoerd Jacob De Vries

**Affiliations:** 1 RPBS platform, Paris, France; 2 LORIA (CNRS, INRIA), University of Lorraine, Nancy, France

**Summary**

 The authors describe the main goals and provide a road-map of the 3D-Bioinfo community, a new Elixir community. A first part states the major contributions and the leading role of many European teams and initiatives in the structural bioinformatics field. Then, the current limitations and challenges faced by this field are discussed, as well as current European initiatives to address them. The core of the paper is the detailed presentation of the goals of the 3D-Bioinfo community, subdivided in activities (or topics) and few main tasks per activity. Details on the implementation of those tasks and on their interconnections are also provided, as well as their complementarity with other European initiatives (especially within ELIXIR).

**Major comment**

 In the paragraph “Develop a knowledge portal to user-friendly bioinformatics and computational tools for modeling conformational flexibility of proteins”, it is surprising to see no mention of any task to define common standards and formats in the description of proteins flexibility.

**Minor comments**

 “However, many challenges still exist. It remains computationally expensive to build 3D models on a proteome-wide scale”

 => Yet this aim is being addressed by the SWISS-MODEL initiative. This assertion should be justified.

 “Community will also undertake dedicated educational, training and outreach efforts to facilitate this, bringing new insights and thus facilitating the development of much needed innovative applications e.g. for human health, drug and protein design.”

 => As I understand it, those educational and training efforts are targeting non-informatician structural biologists, and the idea would be to provide them enough insights in 3D-bioinfo for them to foresee possible applications on their system, and apply tools or seek collaboration in 3D-bioinfo. If the training efforts target young 3D-bioinformaticians, then the link with facilitating applications is unclear. The audience could be mentioned here, for clarity.

 “Structural bioinformatics tools link sequence and structure data to predict protein functional sites”

 => This sounds like a (restrictive) definition of Structural bioinformatics tools. “[Some] structural bioinformatics tools “ would be more correct.

 “As for protein structure prediction, integration of data on sites predicted by different methods will increase both coverage and accuracy.”

 => This assumption could be softened , as the success of this integration is expected but not certain.

 “This enables the design of new experiments to study the function of macromolecules as well as rational design of proteins[,RNA ?] and drugs, to modify their function and properties.”

 “European structure-based tools have facilitated enzyme reaction mechanism studies by chemists and biochemists.“

 => some references would be welcome here

 “The first workshop of the Activity IV is going to take place in May 2020”

 => This should be updated

 “The above mentioned themes will not only form the initial focus for 3D-Bioinfo but the steering committee will actively monitor the emergence of new technologies and/or new research fields relevant for bioinformatics approaches, which then can be fostered further as new activities. “

 => why “will not only form the initial focus”, if the other focuses are supposed to emerge in the future?

**Cosmetic comments**

 [  ] = to add ; { } = to remove

*These are small typo or missing comas that are pretty harmless individually, but some can be misleading. And summed up, they impair a fluid reading of this already quite dense paper.*

 “The technological developments in MX in the previous decade, largely catalysed by the structural genomics initiatives[,] and the on-going revolution in the field of cryo-EM”

 “some of the most critical contributions to building protein 3D models from structural templates of homologous proteins{,} happened in Europe in the 1990s”

 “Importantly[,] European groups have made major contributions to initiatives “

 “In particular, the RNA-Puzzles experiment for evaluation of RNA structure prediction methods, and a series of associated workshops[,] have been introduced in Europe,”

 “(i.e. CASP27, CAMEO28 and CAPRI29,30 [ ) ] for assessment of the prediction “

 “Below we detail{,} the specific thematic aims of each Activity”

 “We will bring together the community experts to define data standards for different types of annotations [,] and integration of these annotations using a community-driven data exchange format will facilitate finding,”

 “Extend the content of OpenEBench by adding {a} knowledge base integrating software tools for modeling the 3D struc-ture of proteins, protein complexes and assemblies.”

 “methods for generating[,?] scoring, and ranking models of protein complexes”

 “Benchmarking studies performed so far [-] including those carried out in the CSAR52 or D3R Grand Challenge53 (...) - rely on datasets of limited size and diversity (…).”

 “Developing benchmarks dataset[s] / Developing [a] benchmarks dataset”

 “In order to foster new developments[,] focused courses/workshops will be organised on such topics”

 “ exploit ELIXIR{s} platforms “

Is the topic of the opinion article discussed accurately in the context of the current literature?

Yes

Are arguments sufficiently supported by evidence from the published literature?

Yes

Are all factual statements correct and adequately supported by citations?

Yes

Are the conclusions drawn balanced and justified on the basis of the presented arguments?

Yes

Reviewer Expertise:

Computational molecular modeling, structural bioinformatics

We confirm that we have read this submission and believe that we have an appropriate level of expertise to confirm that it is of an acceptable scientific standard.
